# Molecular Pathology of Cancer: The Past, the Present, and the Future

**DOI:** 10.3390/jpm11070676

**Published:** 2021-07-19

**Authors:** Leonhard Müllauer

**Affiliations:** Department of Pathology, Medical University Vienna, Waehringer Guertel 18-20, A-1090 Vienna, Austria; leonhard.muellauer@meduniwien.ac.at

Clinical pathology developed from the study of macroscopic organ and tissue changes at autopsies [1]. The first Institutes of Pathology emerged in the first half of the 19th century. The advent of light microscopy in the second half of the 19th century revolutionized clinical pathology. In the 20th century, histological staining techniques were refined, immunohistology and in-situ hybridization methods established, and molecular pathological investigations introduced. The first two decades of the 21st century, however, were shaped by an expansion of molecular pathology.

The diagnosis of neoplastic diseases is the main focus of clinical pathology. Reproducible and internationally comparable diagnostics need standards for the interpretation of morphology, immunohistochemical stains, molecular tests, and nomenclature. The World Health Organization (WHO) and the International Agency for Cancer Research (IARC) established with the aid of international experts a classification of tumors that has meanwhile been published in a series of reference books [2]. In 2001, a paradigm shift occurred in the 3rd edition of the WHO classification of hematopoietic neoplasms “Pathology and Genetics—Tumours of Haematopoietic and Lymphoid Tissues” with the inclusion of genetic findings in tumor classification. This approach has been pursued and expanded ever since—also through the classification of other tumor entities.

Pathology has become more integrated into the clinic over the past decades, in particular through the development of targeted therapies and the associated need to determine predictive biomarkers. First historical examples for the molecular pathological determination of biomarkers for targeted therapies in solid tumors are the *HER2* amplification in breast carcinoma, *KRAS* mutation in colorectal cancer, and *EGFR* mutation in lung adenocarcinoma. Indicative of the proclaimed era of precision medicine is also the formation of molecular tumor boards, in which molecular pathological findings, especially in patients resistant to standard therapy, are discussed.

However, molecular pathological investigations serve not only the purpose of identifying therapy targets or resistance mechanisms but also that of diagnosing and classifying tumors that are characterized by recurrent genetic aberrations. The advancement of molecular pathology has been driven not only by the increase in knowledge derived from cancer research but also through technical innovations, in particular, the development of “next-generation sequencing” (NGS). NGS has from about approximately 2010 gradually and progressively found wider implementation in clinical pathology departments and is at present the workhorse of molecular pathology.

This special issue entitled “Molecular Pathology of Cancer: The Past, the Present, and the Future” encompasses 12 publications from researchers working on a diverse range of neoplastic diseases and addressing various research questions. However, a theme common to most of the contributions is the elucidation of biomarkers for targeted therapies, such as *BRCA 1/2* mutations in ovarian cancer [3] and homologous recombination DNA repair deficiency [4], which are at present amenable to PARP inhibitor therapy. Furthermore, fusions of the *ALK*, *ROS1*, and *NTRK* genes, although overall rare in cancers, are frequently effectively inhibited by targeted drugs and addressed in this compendium for glioma [5] and non-small cell lung cancer [6]. In very rare tumor entities, such as primary diffuse leptomeningeal melanomatosis, with no effective and established standard therapy available, molecular profiling of tumor cells may reveal potential targets for therapy [7]. Pathogenic germline sequence variants are not only a potential cause of cancer development and affect whole families but also constitute targets for therapy, as exemplified for the *BRCA 1/2* [3] and *KIT* genes [8]. Furthermore, certain germline sequence variants may affect clinical outcomes, such as variants of the stromal interaction molecule 1 (STIM1) in breast cancer patients [9]. Molecular pathology may aid in the risk prognostication of malignant diseases and thus facilitate the assignment of patients to different treatment regimens. It thereby complements traditional histopathological diagnoses and parameters such as tumor subtype, tumor differentiation grading, proliferation index, and tumor stage. This is illustrated by Oberndorfer et al. for endometrial cancer, where molecular subgrouping may aid in risk prediction and therapy stratification [10].

The analysis of DNA that is released from dying tumor cells and present in body fluids, the so-called “liquid biopsy,” is an emerging new diagnostic tool in molecular pathology that may aid in the identification of therapeutic targets and resistance mechanisms, in disease monitoring and the detection of minimal residual disease. In patients with brain tumors, the acquisition of diagnostic tumor tissue is often hampered by the risks of biopsy complications and deterioration of patients with advanced disease. Particularly in such scenarios, liquid biopsy could offer an alternative to the analysis of tissue as described by Baumgartner A. et al. and Madlener S. and Gojo J. [7,11]. The increased use of liquid biopsy and the associated employment of ever more sensitive molecular assays for mutation detection pose the difficulty of differentiating tumor-associated mutations from mutations that are mostly derived from blood cells in seemingly healthy patients and increase in frequency with age, a phenomenon termed clonal hematopoiesis of indeterminate potential and representing a potential pre-phase of hematologic neoplasm [12].

Cytology, the morphological analysis of single cells isolated from body fluids, such as pleural effusion or ascites, is the second morphological diagnostic pillar besides histology in clinical pathology. However, the sensitivity of traditional cytology is often limited by the paucity of malignant cells and the difficulty of differentiating them from normal or reactive cells. Grigoriadou et al. present data that support the molecular profiling of malignant pleural effusion with NGS [13].

The further development of molecularly defined personalized medicine poses many challenges to the education and training of physicians and an increasing need to inform and advise patients on molecular testing and the interpretation of results [14].

The future of clinical pathology is morpho-molecular. Traditional histopathological findings will increasingly be combined with molecular pathological results. Nevertheless, it is to be hoped that clinical pathology can again broaden its specialist spectrum beyond the currently dominant tumor pathology and that it perpetuates the great tradition as the “science of diseases.”

## Data Availability

Not applicable.
